# Obacunone Photoprotective Effects against Solar-Simulated Radiation–Induced Molecular Modifications in Primary Keratinocytes and Full-Thickness Human Skin

**DOI:** 10.3390/ijms241411484

**Published:** 2023-07-14

**Authors:** Paula Montero, Maria José Villarroel, Inés Roger, Anselm Morell, Javier Milara, Julio Cortijo

**Affiliations:** 1 Department of Pharmacology, Faculty of Medicine, University of Valencia, 46010 Valencia, Spain; 2 Faculty of Health Sciences, Universidad Europea de Valencia, 46185 Valencia, Spain; 3 Department of Functional Biology and Physical Anthropology, Faculty of Biological Sciences, University of Valencia, 46010 Valencia, Spain; 4 Biomedical Research Networking Centre on Respiratory Diseases (CIBERES), Health Institute Carlos III, 28029 Madrid, Spain; 5 Department of Biochemical Sciences, Faculty of Pharmacy, Charles University, 50005 Hradec Králové, Czech Republic; 6 Pharmacy Unit, University General Hospital Consortium, 46014 Valencia, Spain

**Keywords:** obacunone, photoprotection, full-thickness skin model, photodamage

## Abstract

Solar radiation can cause damage to the skin, leading to various adverse effects such as sunburn, reactive oxygen species production, inflammation, DNA damage, and photoaging. To study the potential of photoprotective agents, full-thickness skin models are increasingly being used as in vitro tools. One promising approach to photoprotection involves targeting the redox-sensitive transcription factor Nrf2, which is responsible for regulating various cellular defense mechanisms, including the antioxidant response, inflammatory signaling, and DNA repair. Obacunone, a natural triterpenoid, has been identified as a potent Nrf2 agonist. The present study aims to evaluate the relevance of full-thickness (FT) skin models in photoprotection studies and to explore the potential photoprotective effects of obacunone on those models and in human keratinocytes. Phenion^®^ full-thickness skin models and keratinocytes were incubated with increasing concentrations of obacunone and irradiated with solar-simulated radiation (SSR). Various photodamage markers were evaluated, including histological integrity, oxidative stress, apoptosis, inflammation, photoaging-related dermal markers, and photocarcinogenesis markers. Increasing doses of SSR were found to modulate various biomarkers related to sun damage in the FT skin models. However, obacunone attenuated cytotoxicity, inflammation, oxidative stress, sunburn reaction, photoaging, and photocarcinogenesis in both keratinocytes and full thickness skin models exposed to SSR. These results suggest that obacunone may have potential as a photoprotective agent for preventing the harmful effects of solar radiation on the skin.

## 1. Introduction

The skin is the largest organ in the human body, and it is the first barrier that protects the body from a wide variety of external environmental stressors [1]. Solar radiation is a significant external agent that can cause damage to the skin [2]. It consists of ultraviolet radiation (UV), visible light, and infrared radiation [3]. Among these, the light that reaches the Earth’s surface is composed of two distinct wavelengths, the most energetic, UVB (290–320 nm), and UVA (320–400 nm), which accounts for approximately 95% of the UV light that reaches the earth surface [4,5]. UVB irradiation causes sunburn, erythema, and DNA damage, which are major risk factors for skin carcinogenesis [6], whereas UVA radiation, due to its higher penetration properties, induces the release of reactive oxygen species (ROS) [7], inflammation, oxidative DNA damage, and photoaging [8]. However, studying these effects in vivo poses technical and ethical challenges. On the other hand, in vitro monolayer cell cultures have limitations in replicating realistic skin physiologic conditions, such as epidermal stratification and cell-to-cell interactions [9].

For these reasons, full-thickness (FT) skin models have been developed. These models are designed to mimic the structure and function of human skin by creating a three-dimensional environment with fully differentiated epidermal layers and the potential for communication between keratinocytes and fibroblasts. The FT skin models have a wide potential to be used as predictive in vitro tools to evaluate the biological events occurring after UV exposure and therefore to be used in photoprotection studies [7].

Although sunscreen is an effective strategy for reducing the damaging effects of solar radiation on the skin, efforts in the industry have shifted towards developing molecular strategies that utilize mechanisms of action beyond photon absorption and reflection [10,11,12]. One promising approach involves targeting the redox-sensitive transcription factor Nrf2 (nuclear factor-E2-related factor 2), which controls different cellular defense mechanisms, such as the antioxidant response, inflammatory signaling, and DNA repair [10]. It has been observed that Nrf2 protects both keratinocytes and skin fibroblasts against the cytotoxic effects of UVA and UVB rays [10,13].

Additional evidence showed that in Nrf2 knockout mice, UVB-induced inflammation was more severe in the absence of Nrf2 compared to wild-type mice [14], while Nrf2 overexpression in skin-derived precursors significantly ameliorated UV-induced damage [15]. These findings highlight the role of Nrf2 in protecting the skin against solar injuries. Consequently, pharmacological modulation of Nrf2 has been considered as a novel approach for skin photoprotection [16,17]. 

Obacunone, a natural triterpenoid limonoid found in citrus and plants of the Rutacea family, has been identified as a potent Nrf2 agonist, and its biological activities have been attributed to its ability to detoxify ROS [18]. Obacunone exerts different pharmacological activities, including antioxidant, anti-inflammatory, antiproliferative, and antitumor activities [19,20]. A recent study reported that obacunone activated the Nrf2 cascade to protect retinal epithelial cells against solar radiation-induced oxidative injury [21]. However, the potential protective effects of obacunone against solar radiation on skin have not been explored. In light of these findings, the present study aims to investigate the biological responses to solar-simulated radiation in a commercial FT skin model and evaluate its relevance for use in photobiological studies. In addition, we will explore the potential photoprotective effects of obacunone on primary human keratinocytes and the FT skin model.

## 2. Results

### 2.1. Evaluation of the Impact of Solar-Simulated Radiation(SSR) on Full-Thickness Skin Models

**Figure 1:**
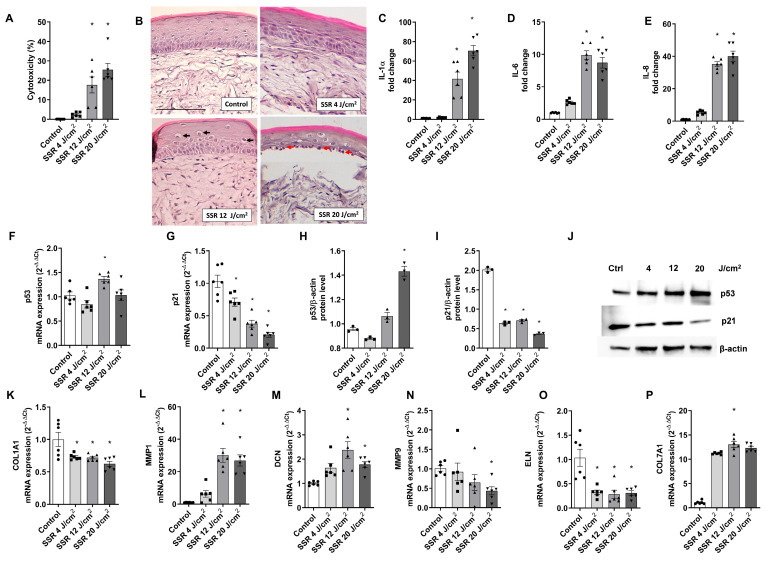
Damaging effects of solar-simulated radiation on a reconstructed full-thickness (FT) human skin. FT skin models were irradiated with 4 J/cm^2^, 12 J/cm^2^, and 20 J/cm^2^ of solar-simulated radiation (SSR). (**A**) LDH cytotoxicity assay. (**B**) Paraffin tissue sections were stained with hematoxylin and eosin. Black arrows indicate localization of vacuolization, and red arrows indicate photodamaged keratinocytes (sunburn cells). Scale bar: 100 μM. (**C**–**E**) IL-1α, IL-6, and IL-8 levels were measured by ELISA. (**F**,**G**) p21 and p53 mRNA levels were measured by real-time PCR. Data are expressed as 2^−ΔΔCt^. (**H**–**J**) p21 and p53 protein levels were analyzed by western blotting. Quantification was performed by densitometry and normalized to β-actin. (**K**–**P**) Decorin (DCN), metalloproteinases 1 and 9 (MMP1, MMP9), elastin (ELN), and collagen type 7 alpha 1 (COL7A1) mRNA levels were measured by real-time PCR. Data are expressed as 2^−ΔΔCt^. All data are expressed as a scatter plot with mean ± standard error of the mean (SEM) of at least two independent experiments (n = 3). Multiple-comparison analysis of variance (ANOVA) was followed by the post hoc Bonferroni test. * *p* < 0.05 vs. control. SSR: Solar-simulated radiation.

With the aim of investigating the harmful effects of solar-simulated radiation (SSR) on full-thickness (FT) skin models, various experiments were performed following exposure of the tissues to increasing doses of SSR. The cytotoxic effects of solar-simulated radiation on the full-thickness skin model were evaluated using the LDH assay. While none of the doses decreased cell viability below 90%, as determined by the MTT assay, increasing doses of SSR led to an increase in LDH release, reaching 25.39 ± 4.011% at the highest dose of 20 J/cm² (Figure 1A). Histological evaluation of the SSR-induced tissue damage in the FT skin model was performed by hematoxylin eosin staining (H&E). Visible morphological modifications were detected at a dose of 12 J/cm^2^ with a loss of cell adhesion in the granulosum and spinosum stratum accompanied by perinuclear vacuolization of keratinocytes (black arrow). Exposure to higher doses of 20 J/cm^2^ induced weakening of the dermo-epidermal junction, the presence of sunburn cells in the basale stratum, characterized by pyknotic nuclei (red arrow), as well as the partial loss of dermal fibroblasts (Figure 1B). Figure 1C–E shows the effects of SSR on the release of inflammatory cytokines. Exposure to SSR induced a significant increase in IL-1α in IL-6 and IL-8 levels at doses of 12 J/cm^2^ and 20 J/cm^2^. 

To evaluate changes in the gene and protein expression of p21 and p53, RT-PCR and western blot analysis were conducted. FT skin models were exposed to increasing doses of SSR; p53 gene expression showed no significant changes (Figure 1F), while p21 expression decreased progressively (Figure 1G). However, a significant protein expression increase was observed in p53 after 20 J/cm^2^ of SSR (Figure 1H), and, similar to the gene expression results, protein expression of p21 decreased significantly at all doses (Figure 1I). Photoaging dermal biomarkers were also explored after SSR exposure by RT-PCR. Upregulated markers were metalloproteinase 1 (MMP1), decorin (DCN), and collagen type 7 alpha 1 (COL7A1) (Figure 1L,M,P), while increasing doses of SSR induced downregulation of metalloproteinase 9 (MMP9), elastin (ELN), and collagen type I alpha 1 (COL1A1) (Figure 1K,N,O). 

To assess the suitability of the FT skin model for photoprotection studies, a correlation analysis was performed to examine the relationship between the SSR dose curve and the analyzed markers. Table 1 shows the Pearson coefficients (rp) for each biomarker. LDH, IL-1α, and IL-8 release, as well as p53 protein expression, showed a very high positive correlation, whereas p21 gene expression showed a very high negative correlation. In addition, IL-6 cytokine levels showed a high positive correlation, and the expression markers COL7A1, GPX1, MMP1, MMP9, and COL1A1 presented a high negative correlation. Finally, ELN and DCN exhibited moderate negative and positive correlation, respectively.

Based on the above results, an intermediate dose was chosen for the subsequent photoprotection experiments on the FT skin model, considering the two highest doses employed in the preliminary studies (12 J/cm^2^ and 20 J/cm^2^). The selected dose was determined to be 18 J/cm^2^. For photoprotection studies with NHEK keratinocytes, a dose-response study was conducted to analyze cytotoxicity and IL-8 release, in the range of 1–10 J/cm^2^ (). In that case, a dose of 5 J/cm^2^ was chosen for the photoprotection studies in NHEK cells. 

### 2.2. Obacunone Attenuates SSR-Induced Cytotoxicity and Tissue Damage in NHEK Cells and in the FT Skin Model

**Figure 2:**
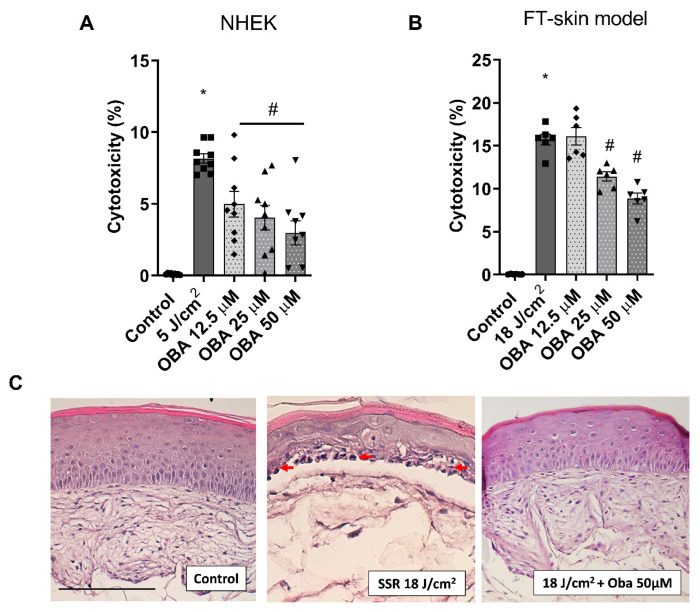
Obacunone attenuates UVR-induced cytotoxicity in NHEK cells and a FT skin model and ameliorates tissue damage and sunburn reaction in the FT skin model. (**A**) NHEK cells pretreated for 24 h with obacunone (12.5 µM, 25 µM, and 50 µM) and subjected to 5 J/cm^2^ SSR; 24 h after irradiation, the LDH cytotoxicity assay was performed. (**B**) FT skin models pretreated for 24 h with obacunone (12.5 µM, 25 µM, and 50 µM) and subjected to 18 J/cm^2^ of SSR; 24 h after irradiation, the LDH cytotoxicity assay was performed. (**C**) FT skin models pretreated for 24 h with obacunone 50 µM and subjected to 18 J/cm^2^ of SSR. Paraffin tissue sections were stained with hematoxylin and eosin. Red arrows indicate photodamaged keratinocytes (sunburn cells). Scale bar: 100 μM. All data are expressed as a scatter plot with mean ± standard error of the mean (SEM) of at least two independent experiments (n = 3). Multiple-comparison analysis of variance (ANOVA) was followed by the post hoc Bonferroni test. * *p* < 0.05 vs. control. # *p* < 0.05 vs. SSR stimulus. SSR: Solar-simulated radiation. OBA: obacunone.

Exposure to solar-simulated radiation in NHEK cells and FT skin models at doses of 5 J/cm^2^ and 18 J/cm^2^, respectively, induced an increase in LDH release. Obacunone significantly attenuated the LDH release in NHEK cells at all doses studied (Figure 2A) and at doses of 25 and 50 µM in the FT skin model (Figure 2B). Figure 2C shows the histological changes in the FT skin model. Pre-treatment with obacunone diminished the formation of sunburn cells (red arrows) and protected from the destruction of the dermal compartment.

### 2.3. Obacunone Protective Effects against SSR-Induced Apoptosis in NHEK Cells and in the FT Skin Model

**Figure 3:**
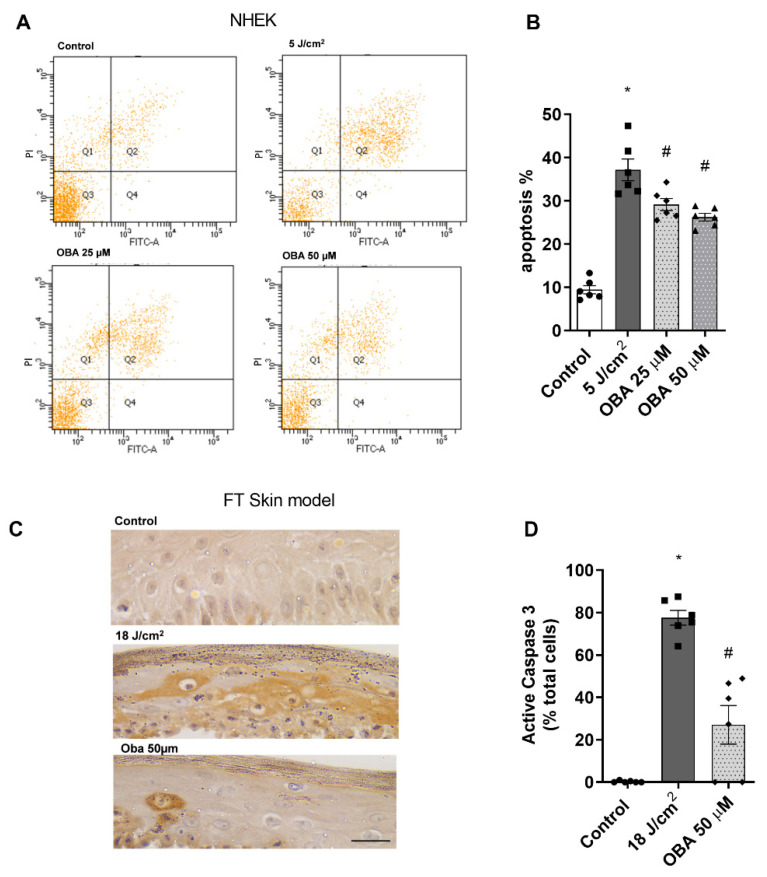
Obacunone attenuates SSR-induced apoptosis in NHEK cells and in a FT skin model. NHEK cells were pretreated for 24 h with obacunone (25 µM and 50 µM) and were then subjected to 5 J/cm^2^ of SSR. After irradiation, apoptosis was measured by flow cytometric analysis. (**A**) Representative plots for each condition are displayed. (**B**) Apoptosis plots were analyzed by FlowJo software (Version number 10, TreeStar Inc., Ashland, OR, USA). Results are expressed as the mean apoptosis percentage of annexin-positive and propidium iodide-negative cells plus double-positive cells. (**C**) FT skin models were pretreated for 24 h with obacunone 50 µM and subjected to 18 J/cm^2^ of SSR; 24 h after irradiation, reconstructs were harvested and processed for immunohistochemistry of cleaved procaspase 3. Scale bar: 30 μm. (**D**) The bar graph displays quantitative analysis of active caspase-3 marked keratinocytes (percentage of positive cells per high power field). All data are expressed as a scatter plot with the mean ± standard error of the mean (SEM) of at least two independent experiments (n = 3). Multiple-comparison analysis of variance (ANOVA) was followed by the post hoc Bonferroni test. * *p* < 0.05 vs. control. # *p* < 0.05 vs. SSR stimulus. OBA: obacunone.

As sunburn cells precede cellular apoptosis, the protective effect of obacunone against SSR-induced cell apoptosis was studied by flow cytometry in NHEK keratinocytes and by immunohistochemical visualization of cleaved procaspase 3 (active caspase 3) in the FT skin models. SSR increased the percentage of apoptotic keratinocytes, an effect that was significantly prevented by obacunone treatment at 25 µM and 50 µM (Figure 3A,B). Likewise, in the FT skin model, irradiation increased active caspase-3 staining, while tissues treated with obacunone had decreased active caspase-3 (Figure 3C,D).

### 2.4. Obacunone Slightly Ameliorates SSR-Induced Inflammation in NHEK Cells and in the FT Skin Model

**Figure 4:**
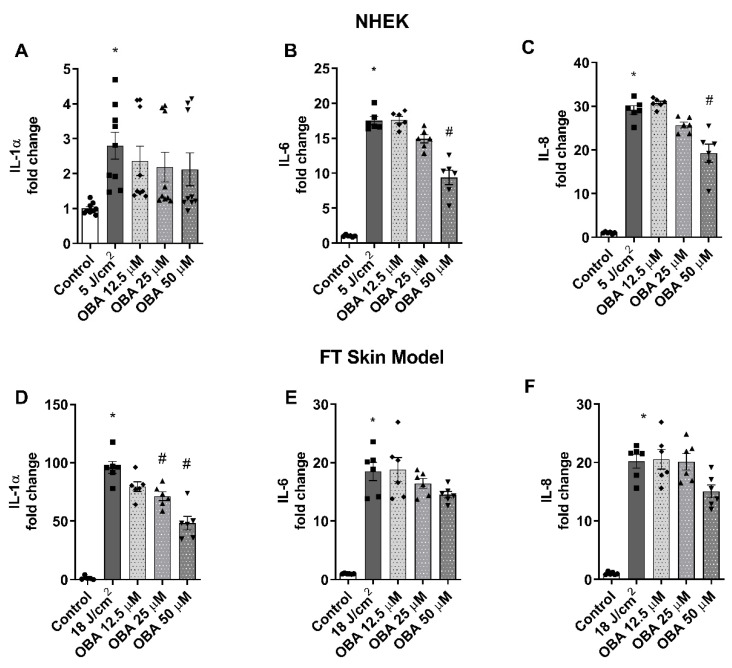
Obacunone has a slight effect in preventing cytokine release induced by SSR in NHEK cells and a FT skin model. (**A**–**C**) NHEK cells were pretreated for 24 h with obacunone (12.5 µM, 25 µM, and 50 µM) and subjected to 5 J/cm^2^ solar-simulated radiation. (**D**–**F**) FT skin models were pretreated for 24 h with obacunone (12.5 µM, 25 µM, and 50 µM) and subjected to 18 J/cm^2^ of solar-simulated radiation. 24 h after irradiation, IL-1α, IL-6, and IL-8 levels were measured by ELISA. All data are expressed as a scatter plot with mean ± standard error of the mean (SEM) of at least two independent experiments (n = 3). Multiple-comparison analysis of variance (ANOVA) was followed by the post hoc Bonferroni test. * *p* < 0.05 vs. control. # *p* < 0.05 vs. SSR stimulus. OBA: obacunone.

**Figure 5:**
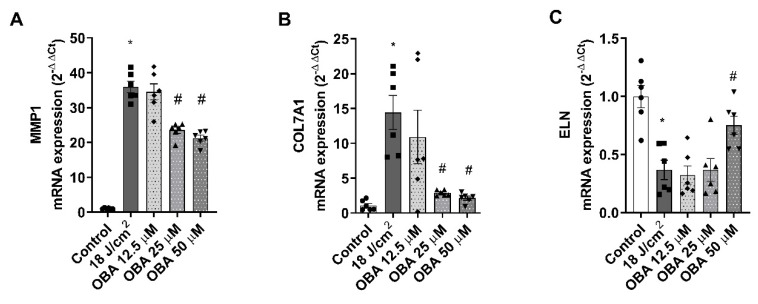
Obacunone attenuates gene expression modulation of photoaging biomarkers in a FT skin model. (**A**–**C**) FT skin models were pretreated for 24 h with obacunone (12.5 µM, 25 µM, and 50 µM) and subjected to 18 J/cm^2^ of SSR; 24 h after irradiation, metalloproteinases 1 (MMP1), elastin (ELN), and collagen type 7 alpha 1 (COL7A1) mRNA levels were measured by real-time PCR. Data are expressed as 2^−ΔΔCt^. All data are expressed as a scatter plot with mean ± standard error of the mean (SEM) of at least two independent experiments (n = 3). Multiple-comparison analysis of variance (ANOVA) was followed by the post hoc Bonferroni test. * *p* < 0.05 vs. control. # *p* < 0.05 vs. SSR stimulus. OBA: obacunone.

The effects of obacunone in preventing the release of inflammatory cytokines was evaluated in NHEK cells and in the FT skin model. Exposure to SSR induced a significant release of IL-1α in IL-6 and IL-8 in all cases. Treatment with obacunone in NHEK keratinocytes induced a tendency to decrease the cytokine release, which was significant only at the highest concentration used (50 µM) for IL-6 and IL-8 cytokines (Figure 4B,C) but was not significantly reduced in the case of IL-1α (Figure 5A). Similar to these responses, obacunone slightly prevented the increase in these cytokines in the FT skin model. Although at 50 µM concentration the cytokine levels were diminished, these decreases were only statistically significant for IL-1α (Figure 4D–F).

### 2.5. Obacunone Attenuates Modulation of Photoaging Biomarkers in a FT Skin Model

Gene expression analysis was performed by PCR to evaluate the modulation of photoaging dermal biomarkers after SSR exposure in the FT skin model. While treatment with obacunone did not prevent the modulation of DCN, COL1A1, and MMP9, obacunone at 25 µM and 50 µM significantly attenuated the upregulation of MMP1 and COL7A1 (Figure 5A,B) and significantly prevented ELN downregulation at 50 µM (Figure 5C).

### 2.6. Obacunone Prevents SSR-Induced Modulation of Photocarcinogenesis Biomarkers in a FT Skin Model

**Figure 6:**
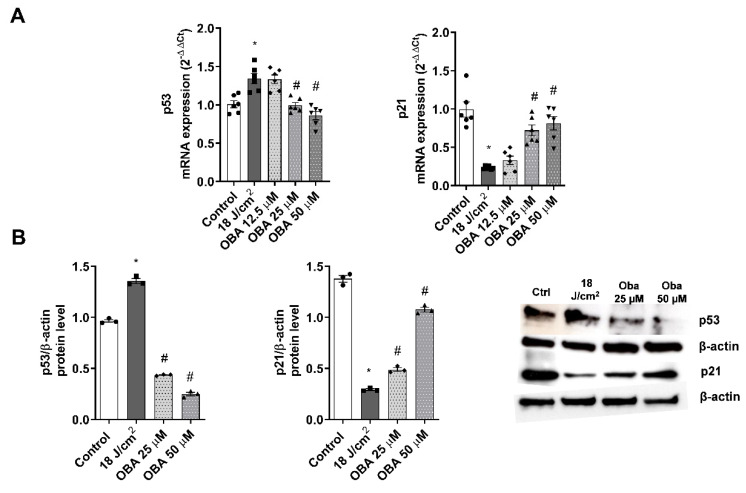
Obacunone prevents SSR-induced p53 and p21 gene and protein expression modulations in a FT skin model. FT skin models were pretreated for 24 h with obacunone (12.5 µM, 25 µM, and 50 µM) and subjected to 18 J/cm^2^ of SSR. (**A**) 24 h after irradiation, p21 and p53 mRNA levels were measured by real-time PCR. Data are expressed as 2^−ΔΔCt^. (**B**) p21 and p53 levels were analyzed by Western blotting. Quantification was performed by densitometry and normalized to β-actin. Data are expressed as 2^−ΔΔCt^. All data are expressed as a scatter plot with mean ± standard error of the mean (SEM) of at least two independent experiments (n = 3). Multiple-comparison analysis of variance (ANOVA) was followed by the post hoc Bonferroni test. * *p* < 0.05 vs. control. # *p* < 0.05 vs. SSR stimulus. OBA: obacunone.

To evaluate the variations in the gene and protein expression of p21 and p53, RT-PCR and western blot analysis were performed. After exposing the FT skin models to increasing SSR, p53 increased expression while p21 was downregulated. Pre-treatment with obacunone at 25 and 50 µM significantly attenuated modulation of both p21 and p53 markers at the gene and protein expression levels (Figure 6A,B).

### 2.7. Obacunone Ameliorates SSR-Induced Oxidative Injury in NHEK Cells and in the FT Skin Model

**Figure 7:**
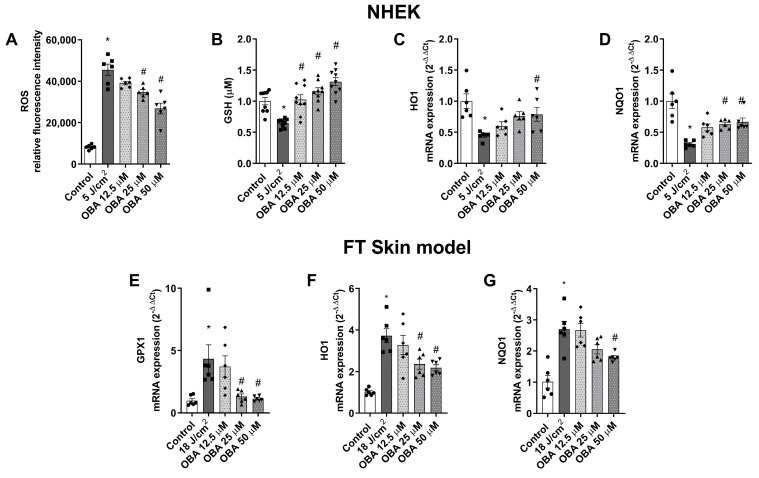
Obacunone ameliorates SSR-induced oxidative injury in NHEK cells and in a FT skin model. NHEK cells were pretreated for 24 h with obacunone (12.5 µM, 25 µM, and 50 µM) and subjected to 5 J/cm^2^ of SSR. (**A**) After irradiation, quantification of reactive oxygen species (ROS) levels was measured by the CM-H_2_DCFDA assay. Data are expressed as reactive oxygen species (ROS) DCF relative fluorescence units. (**B**) Total cellular glutathione levels (GSH) were measured by the GSH-Glo™ Glutathione Assay kit. (**C**,**D**) 24 h after irradiation, heme-oxygenase (HO1) and NADPH quinone dehydrogenase (NQO1) mRNA levels were measured by real-time PCR. Data are expressed as 2^−ΔΔCt^. (**E**–**G**) FT skin models were pretreated for 24 h with obacunone (12.5 µM, 25 µM, and 50 µM) and subjected to 18 J/cm^2^ of SSR; 24 h after irradiation, glutathione peroxidase 1 (GPX1), HO1 and NQO1 mRNA levels were measured by real-time PCR. Data are expressed as 2^−ΔΔCt^. All data are expressed as a scatter plot with mean ± standard error of the mean (SEM) of at least two independent experiments (n = 3). Multiple-comparison analysis of variance (ANOVA) was followed by the post hoc Bonferroni test. * *p* < 0.05 vs. control. # *p*< 0.05 vs. SSR stimulus. OBA: obacunone.

**Figure 8:**
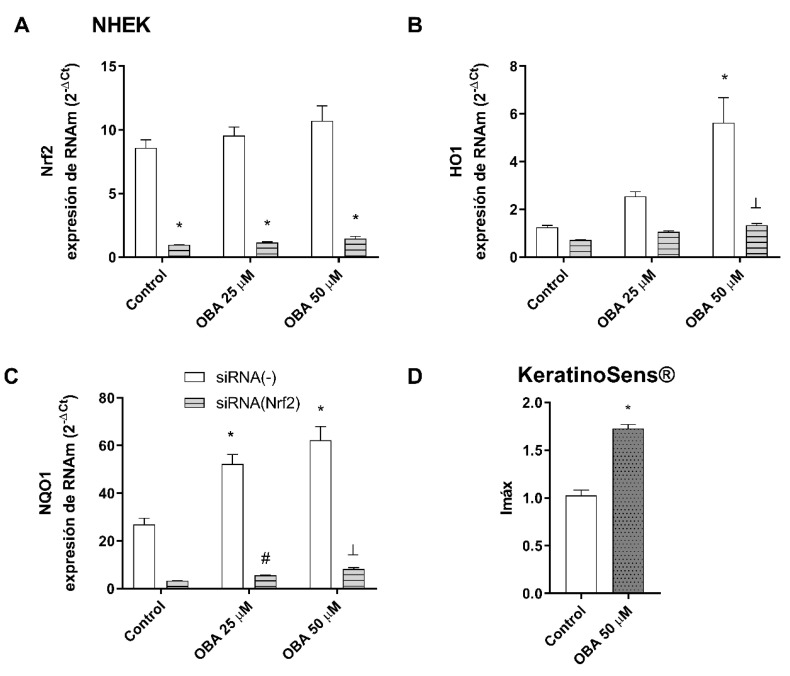
Obacunone activates Nrf2 signaling cascade in NHEK cells. (**A**–**C**) NHEK keratinocytes were transiently transfected with control siRNA(−) or siRNA(Nrf2) and incubated for 24 h with obacunone at 25 µM and 50 µM. Nrf2, heme-oxygenase (HO1), and NADPH quinone dehydrogenase (NQO1) mRNA levels were measured by real-time PCR. Data are expressed as 2^−ΔCt.^ * *p* < 0.05 vs. siRNA(−) control. # *p* < 0.05 vs siRNA(−) OBA 25. ⊥ *p* < 0.05 vs siRNA(−) OBA 50. (**D**). Keratinosens cell line was incubated with 50 µM obacunone, and the ARE-Nrf2 luciferase test was performed. Data are expressed as induction of luciferase activity (Imax). All data are expressed as a scatter plot with mean ± standard error of the mean (SEM) of at least two independent experiments (n = 3). Multiple-comparison analysis of variance (ANOVA) was followed by the post hoc Bonferroni test. * *p* < 0.05 vs. control. # *p* < 0.05 vs. SSR stimulus. OBA: obacunone.

The protective effects of obacunone against SSR-induced oxidative stress response was analyzed in NHEK and in the FT skin model by measuring intracellular ROS production, GSH content, and the gene expression of glutathione peroxidase 1 (GPX1), heme-oxygenase (HO1), and NADPH quinone dehydrogenase (NQO1). In NHEK keratinocytes, incubation with obacunone at the highest dose of 50 µM significantly prevented the SSR-induced release of ROS (Figure 7A). Moreover, obacunone at all doses prevented oxidative damage by increasing GSH content in NHEK, even above basal levels (Figure 8B). Additionally, SSR induced the downregulation of antioxidant genes NQO1 and HO1, but obacunone significantly prevented this effect (Figure 7C,D). In the FT skin model, obacunone prevented the GPX1 increase induced by exposure to SSR (Figure 7E). Furthermore, at doses of 25 µM and 50 µM, obacunone significantly prevented the upregulation of NQO1 and HO1 (Figure 7F,G).

### 2.8. Obacunone Activates Nrf2 Signaling Cascade in NHEK Cells

NHEK keratinocytes were transiently transfected with siRNA (Nrf2) to reduce Nrf2 expression. As seen in Figure 8A, Nrf2 silencing was confirmed. The siRNA-Nrf2 transfection abrogated the effects of obacunone in increasing HO1 and NQO1 gene expression (Figure 8B,C). To support these results, the Keratinosens assay was performed. Keratinosens are transfected cells that express the luciferase gene under the control of the Antioxidant Response Element (ARE). Therefore, when the antioxidant Nrf2 response is activated, it leads to an increase in the luciferase activity of these cells. The results depicted in Figure 8D demonstrate that the incubation of Keratinosens cells with 50 µM obacunone resulted in a significant increase in luciferase activity.

## 3. Discussion

The exposure of skin to solar irradiation is one of the principal causes that leads to the development of cutaneous disorders such as sunburn, carcinogenesis, oxidative stress, inflammation, and photoaging [6,7,8]. As the study of the clinical effects of solar radiation on skin has limitations due to technical and ethical reasons in vivo, different organotypic skin reconstructs have been developed by the industry as an alternative that mimics skin architecture and physiology. The aim of this study was to assess the effectiveness of the Phenion^®^-FT skin model as a tool for evaluating the biological effects of solar exposure and identifying potential biomarkers for use in photoprotection studies. We also examined the protective effects of obacunone, a Nrf2 activator, against solar-simulated radiation (SSR), using both the Phenion^®^-FT skin model and NHEK keratinocytes. 

First, we analyzed the biological effects of solar-simulated radiation (SSR) on the FT skin models. Our findings confirmed that increasing the dose of SSR radiation resulted in modulation of various biomarkers related to sun damage. Specifically, exposure to SSR induced the modulation of biomarkers related to cytotoxicity, inflammation, sunburn reaction, photoaging, and photocarcinogenesis. Our findings are consistent with previous studies that demonstrated that exposure to isolated UVA and UVB radiation can induce the release of IL-6 and IL-8 [22] and IL-1 and IL-6 [23], respectively, while IL-8 is known to increase in human skin after exposure to UV radiation [24]. The appearance of sunburn cells, which is related to keratinocytes apoptosis and the loss of dermal fibroblasts, is also a hallmark of sun damage [9,25,26]. P53 and p21 modulation have been recognized as significant features of photocarcinogenesis. Under normal conditions, following UV exposure, the p53 protein is upregulated in the epidermis, subsequently inducing p21 expression and leading to cell cycle arrest [27]. However, various responses have been reported in the literature, likely attributed not only to the different systems analyzed (such as monolayer cultures, epidermal equivalents, and full thickness equivalents) but also to variations in the dosage and time points assessed. In our study, we observed an elevation in p53 levels and a decrease in p21 expression after SSR exposure. It has been demonstrated that, while low doses of UVB induce p21, higher doses can block and even suppress its expression. These findings indicate that low doses promote growth arrest and repair mechanisms, whereas high doses result in apoptosis [28,29]. Studies conducted on monolayer cultures have revealed contrasting responses. For instance, primary keratinocytes showed increased p53 levels following UV exposure [30], whereas HaCat keratinocytes irradiated with UVB exhibited unaltered p53 levels and decreased p21 expression [31]. Conversely, another investigation using HaCat cells and UVB reported a down-regulation of both p21 and p53 [32]. Studies involving skin models have limited available data. Marot et al. described a bell-shaped expression pattern of p53 in reconstructed skin models following UV light exposure [33], in line with the results obtained in our study. Based on this evidence, we propose the hypothesis that, at the dosage employed in our study, SSR increased the protein content of p53, while reduced levels of p21 could lead to cellular apoptosis. 

Next, a series of photoaging biomarkers was evaluated. The results showed modulation of MMP1, DCN, COL7A1, MMP9, ELN, and COL1A1, consistent with the results obtained by Meloni et al. in a study involving skin tissues exposed to UVA radiation at different time points from those employed in our investigation [8]. In addition, some of these responses were similar to those documented in human skin following UVB radiation, such as the modulation of MMP1 [34] and MMP9 [35], which contribute to the degradation of collagen and elastin fibers [36], the key components of the photoaging process. Moreover, we assessed the correlation between all the biomarkers examined and the doses of SSR administered, revealing moderate to very high Pearson coefficients (rp). To date, the Phenion^®^-FT skin model has been used by Meloni et al. to investigate the gene expression profile in response to UVA irradiation under different conditions than those employed in this study [8] and by Pinto et al. to evaluate topical application of sunscreen on an aged model [37]. Therefore, to our knowledge, our investigation represents the first comprehensive photobiological study of SSR-related biomarkers using the Phenion^®^-FT skin model.

As pharmacological modulation of Nrf2 has been considered as a novel approach for skin photoprotection [16], we evaluated the protective effects of the Nrf2 activator obacunone. Obacunone was able to attenuate cytotoxicity in NHEK cells and in the FT model. Similarly, there are other Nrf2 activators that have been documented in the scientific literature for their ability to confer protection against the cytotoxicity caused by UVA and UVB irradiation in both keratinocytes and fibroblasts [10,13]. Cellular apoptosis and histological evaluations were also investigated. In the FT skin model, treatment with obacunone resulted in reduced formation of sunburn cells and protected against destruction of the dermal compartment. Obacunone also prevented apoptosis and decreased activation of caspase 3 in NHEK cells and the FT skin model, respectively. Caspase-3 activation and induction of apoptosis associated with UVR damage constitutes the last line of defense, eliminating damaged cells following p53 activation [38]. Similar findings have been reported by other authors. For instance, Nrf2 overexpression ameliorates the structural derangement induced by UV, the Nrf2 inducer sulforaphane reduces apoptosis rates and sunburn reaction after UVB irradiation, and the Nrf2-inducer tanshinone prevents UVB-induced activation of caspase 3 in reconstructed human skin [14,15,16]. The protective action of obacunone against SSR-induced p53 and p21 modulations was also assessed in this study. We present novel findings that demonstrate the mitigation of both markers through pre-treatment with obacunone, as evidenced by alterations in gene and protein expression levels.

Regarding inflammation, obacunone showed a tendency to reduce the release of inflammatory cytokines. However, the anti-inflammatory effects were more prominent at the highest doses. It has been observed that in Nrf2 knockout mice, the inflammatory response induced by UVB was reduced in comparison to the wild-type mice [14,39]. Further, administration of the Nrf2 inducer sulforaphane suppresses UVB-induced acute inflammation and sunburn reaction in murine skin; its effects have been attributed not only to its Nrf2 modulation but also to its inhibition of AP-1 [14]. However, our results in vitro show that the effects induced by pharmacological modulation of Nrf2 are not as strong as the genetic modulation. We speculate that incubation with higher obacunone doses or increasing the exposition time will induce a more sustained effect in protecting from inflammation.

As obacunone is known for its antioxidant activities [18], we explored its potential to reduce the oxidative response induced by SSR by analyzing ROS release as well as its ability to increase the contents of the tripeptide glutathione (GSH) in NHEK cells. Obacunone proved to protect against both oxidative responses. Similarly, other authors have shown protective activities against UVB-mediated oxidative stress in other Nrf-2 activators [40]. It is also worth mentioning that the modulation of the Nrf2 response antioxidant genes was different between NHEK cells and FT tissues. The differential response of these genes upon solar stimulation has been explained by Ryšavá et al. [41], who found that in NHEK cells, NQO1 and HO1 levels change with different doses of UVA and with the incubation time after exposure. Results from an in-house reconstructed model showed up-regulation of the expression of HO1 and NQO1 two to six hours after UVA1 exposure [42]. In another reconstructed model, exposure to UV radiation induced upregulation in the early hours post exposure and reductions at 24 h, with different responses amongst skin fibroblasts and keratinocytes [43]. Further, it has been shown that low-dose UVB exposure activates Nrf2 and up-regulation of the antioxidant response element (ARE) gene expression; however, high UVB doses lead to the nuclear exclusion of Nrf2 and down-regulation of gene expression [14]. This could explain the differential effects on genetic regulation, as it appears that different sources of light, doses, and evaluation times after exposure affect responses [40]. As previous studies have demonstrated that dermal fibroblasts are more prone to exhibit increases in HO1 and NQO1 expression compared to epidermal keratinocytes [23,43], our hypothesis regarding the differential responses observed between the monolayer and the FT skin model involves the presence of both fibroblasts and keratinocytes in these models. Therefore, these models show the shared response of both cell types but also highlights the influence of complex intercommunications between them. Furthermore, glutathione peroxidase GPX1 was evaluated in the FT skin model due to its significant role as an antioxidant defense system in the skin. In human volunteers, a decrease in GPX activity has been observed after UV irradiation [44], but it was found to increase after 48 h [45]. However, similar to other antioxidant genes, diverse responses have been reported in the literature. For instance, in dermal fibroblasts exposed to UVA, GPX activity was found to increase [46], and in a FT skin model, gene expression of GPX was upregulated [8]. On the other hand, a separate study indicated a reduction in the protein content of GPX after UVB irradiation [47], suggesting that different wavelengths also influence the response. In murine skin exposed to UVA/UVB radiation, GPX activity was only slightly reduced immediately after irradiation but increased after 3 h [45]. We observed an expected increase in GPX gene expression after SSR, which was prevented by obacunone treatment. It is important to note that, based on previous knowledge, one would expect obacunone to further increase GPX1 expression. We formulated several hypotheses to explain this finding. The significant increase in GPX1 expression is thought to occur as a defense mechanism against the SSR insult. However, it should be acknowledged that the chosen dose for the FT skin model in this study is considered high. Consequently, obacunone treatment may either have prevented an excessive response, or the duration of incubation with obacunone might not have been sufficient to exert its full effect. Another less likely scenario would be that obacunone could inhibit specific UV-induced signaling pathways that are necessary for the upregulation of GPX expression, utilizing mechanisms distinct from Nrf2 activation. Nonetheless, it is worth considering that higher doses or prolonged exposure to obacunone might have contrasting effects on GPX expression. To fully comprehend these responses, additional experiments should be conducted to analyze different doses of SSR and evaluate GPX expression at various time points. It is important to consider that the antioxidant response is highly influenced by early or late responses. Therefore, caution should be exercised when interpreting the antioxidant results in the FT skin models. Further research is needed to fully understand the complex interplay between solar exposure, antioxidant genes, and obacunone.

Finally, although obacunone was not able to prevent the modulation of all the photoaging biomarkers, our study is the first to provide evidence of its beneficial effects on the changes in MMP1, COL7A1, and ELN. We also investigated the underlying mechanisms by performing Nrf2 silencing and keratinosense assays, which revealed that obacunone’s protective antioxidant effects on keratinocytes are likely mediated by the Nrf2 signaling cascade, as previously demonstrated in retinal cells [21].

The results of our study confirm that FT skin models are a reliable tool for investigating photobiological alterations on skin and are an excellent resource for studying photoprotective agents. Given the increasing awareness of the harmful effects of solar radiation on skin, there is a growing need for effective photoprotective agents. 

Our findings demonstrate that obacunone can protect both keratinocytes and FT skin models against solar-induced damage, thereby indicating that Nrf2 activation is a promising target for addressing skin alterations caused by solar radiation. However, obacunone was not able to provide complete protection against all the evaluated markers. Therefore, further studies are necessary to elucidate the molecular mechanism underlying the photoprotective effects of obacunone in order to fully comprehend its potential and optimize its application in skincare strategies.

## 4. Material and Methods

### 4.1. Cell Culture and Treatment

Normal human epidermal keratinocytes (NHEK) (C-12005, PromoCell, Heidelberg, Germany) NHEK were cultured in keratinocyte growth medium-2 (KGM-2), supplemented with SupplementMix and CaCl_2_ (60 μM) (Promocell, Heidelberg, Germany). Reconstructed human skin Phenion^®^ full-thickness (FT) was produced by Henkel (Dusseldorf, Germany) and cultivated according to supplier instructions using the air–liquid interface medium (ALI-CM, Henkel, Dusseldorf, Germany).

For the SSR irradiation procedure, tissues were placed in PBS and irradiated with increasing doses of 4 J/cm^2^, 12 J/cm^2^, and 20 J/cm^2^. Following irradiation, the skin tissues were immediately transferred to fresh ALI medium and recovered in an incubator (37 °C, 5% CO_2_) for 24 h. Obacunone (AI3-37934, CCRIS 8657), 99.85% purity, was purchased from SelleckChem (Shanghai, China). For the photoprotection experiments, obacunone at concentrations of 12.5 µM, 25 µM, and 50 µM was incubated in NHEK cells and Phenion^®^-FT tissues for 24 h. After incubation, both models were irradiated with SSR at 5 J/cm^2^ and 18 J/cm^2^. The SSR source was the Sun Simulator SOL 500 (Dr. Hönle, Hönle GmbH, Martinsried, Germany), with a spectral range corresponding to natural sunlight combined with a filter (H2,). The radiation efficiency of SOL units in the ultraviolet and visible range (295–780 nm) is close to 44%. Technical specifications of the solar unit can be found at (https://www.hoenle.com/products/solar-simulation-systems-and-light-fixtures/sol-light-fixtures, accessed on 5 July 2023). The UV output was measured by a UV-meter (Dr. Hönle, Martinsried, Germany). Test evaluations were performed 24 h after SSR exposure. 

### 4.2. Cytotoxicity Testing

Twenty-four hours after irradiation, culture mediums were collected and the cytotoxicity assay was performed by measuring lactate dehydrogenase (LDH) release in the medium using the commercially available LDH cytotoxicity assay kit (Thermo Fisher Scientific, Madrid, Spain), following the manufacturer’s instructions. Absorbance was measured at 490 nm using the plate reader Infinite M200 (Tecan Group Ltd., Männedorf, Switzerland). LDH contents were normalized to the maximum LDH release. 

### 4.3. Histological and Immunohistochemical Analysis

Twenty-four hours after irradiation, the Phenion^®^-FT tissues were fixed in 4% buffered formaldehyde solution, dehydrated in an alcohol gradient, and embedded in paraffin for sectioning. Sections of 5 µM were stained with hematoxylin and eosin (H&E) for histological evaluation. For immunohistochemical analysis, tissue sections were incubated overnight with the antibody cleaved caspase 3 (Asp175; 9664, Cell Signaling, Madrid, Spain). Master Polymer Plus detection system peroxidase (Ref: MAD-000237QKA; master diagnóstica, Granada, Spain) was used for immunostaining development. The non-immune IgG isotype control was used as the negative control for all samples.

### 4.4. Cytokine Determination by Enzyme Linked Immunosorbent Assay (ELISA)

Twenty-four hours after irradiation, culture mediums were collected, and IL-8, IL-6, and IL-1α cytokine levels were analyzed using commercially available Quantikine^®^ ELISA kits (R&D Systems, Madrid, Spain) according to the manufacturer’s protocol. Results are expressed as fold change relative to control.

### 4.5. Real Time RT-qPCR and siRNA Experiments

Twenty-four hours after irradiation, NHEK cells and Phenion^®^-FT tissues were harvested, and total RNA was extracted using the KingFisher Duo Prime automated extractor (Thermo Fisher Scientific, Madrid, Spain) with the MagMAX™ 96 Total RNA Isolation Kit (Thermo Fisher Scientific, Madrid, Spain) following the manufacturer’s instructions. Reverse transcription was performed in 500 ng of total RNA with the Takara PrimeScript RT Reagent kit (Takarabio, Shiga, Japan). The obtained cDNA was amplified with primers and probes predesigned by Applied Biosystems in a QuantStudio™ 5 Real-Time PCR System, using universal master mix (Applied Biosystems, Thermo Fisher Scientific, Madrid, Spain). The probes used were collagen type I alpha 1 (COL1A1, Hs00164004_m1), collagen type 7 alpha 1 (COL7A1, Hs00164310_m1), decorin (DCN, Hs00754870_s1), elastin (ELN, Hs00355783_m1), metalloproteinase 1 (MMP1, Hs00899658_m1), metalloproteinase 9 (MMP9, Hs00234579_m1), glutathione peroxidase 1 (GPX1, Hs00829989_gH), Heme-oxygenase (HO1, Hs01110250_m1), NADPH quinone dehydrogenase (NQO1, Hs00168547_m1), nuclear factor erythroid 2-related factor 2 (NRF2, Hs00975961_g1), p21 (Hs01040810_m1), and p53 tumor suppressor protein (p53, Hs01034249_m1). Expression of the target gene was expressed as the fold change relative to β-actin (Hs01060665_g1) expression as an endogenous control. The mean value of the replicates for each sample was expressed as the cycle threshold (Ct), and the gene expression level was calculated as the difference (ΔCt) between the target gene Ct value and the β-actin Ct value. The fold changes in the target gene mRNA levels were designated 2^−ΔΔCt^.

Small interfering RNA (siRNA) experiments were carried out in NHEK cells. The scrambled siRNA control (siRNA (−)) (ref. 4390843) and Nrf2 gene-targeted siRNA (siRNA-Nrf2) (ref.107966) were purchased from Ambion (Huntingdon, Cambridge, UK). Cells were transfected with siRNA (50 nM) in serum and antibiotic-free medium. After 6 h, Transiently silenced NHEK were incubated with obacunone for 24 h. The transfection reagent used was lipofectamine 2000 (11668-027, Invitrogen, Paisley, UK) at a final concentration of 2 μg/mL. 

### 4.6. Western Blotting Analysis

Twenty-four hours after irradiation, the protein content from Phenion^®^-FT tissues was quantified using the BCA Protein Assay Kit (Thermo Fisher Scientific, Madrid, Spain). A total of 20 μg of denatured proteins was loaded into Mini-PROTEAN^®^ polyacrylamide gels TGX ™ (Bio-Rad, Madrid, Spain) by application of 150 V for 1 h. Proteins were transferred to a nitrocellulose membrane Trans-Blot^®^ Turbo™ Transfer Pack using the Trans-Blot^®^ Turbo™ Transfer System (Bio-Rad Laboratories, Watford, UK). Then, membranes were incubated with 5% bovine serum albumin (BSA) for 2 h and labeled overnight at 4 °C with the antibodies p21 (NB100-1941, Novus Biologicals, Centennial, CO, USA) and p53 (18032, Cell Signaling, Danvers, MA, USA). Signal visualization of proteins was carried out by incubating the membranes with chemiluminescence reagents (ECL Plus; Amersham GE Healthcare, Saint Giles, UK). Densitometry of films was performed using the Image J 1.42q software. Results of the target protein expression are expressed as the percentage of the densitometry of the endogenous control β-actin. 

### 4.7. ROS DCF Fluorescence Measurement of Reactive Oxygen Species

NHEK cells were cultured in a dark 96-well culture plate with a transparent bottom. After obacunone treatment for 24 h, cells were washed twice with PBS and incubated for 30 min with 2 μM CM-H_2_DCFDA. Then, cells were irradiated, and fluorescent intensity was measured using a microplate spectrophotometer (Victor 1420 Multilabel Counter, PerkinElmer, Madrid, Spain) at excitation and emission wavelengths of 485 and 528 nm. Results were expressed as ROS fluorescence intensity, which indicates DCF fluorescence in relative fluorescence units.

### 4.8. GSH

NHEK keratinocytes were seeded in a dark 96-well plate and treated for 24 h with obacunone. Then, cells were irradiated and washed with phosphate buffer. GSH content was measured with the GSH-Glo™ Assay kit (Promega; Fitchburg, WI, USA) following the manufacturer’s instructions. Luminescence was measured with the luminometer (LUMIstar Omega, BMG Labtech, Ortenberg, Germany), and the results are displayed as GSH µM content. 

### 4.9. Apoptosis

NHEK cells were seeded on 96-well plates and incubated with obacunone for 24 h. Then, cells were irradiated, and 1 h after irradiation, apoptosis was measured using a commercially available Annexin V-FITC apoptosis detection kit (ab14085, Abcam, Cambridge, UK). Cells were detached and collected along with the supernatant and incubated with annexin VFITC 3 μg/mL for 15 min. Then, annexin V binding buffer was added, and prior to flow cytometric analysis, propidium iodide was added at 5 μg/mL. Flow cytometric analysis was performed by a BD LSRFortessa™ X-20 flow cytometer (BD Biosciences; San Jose, CA, USA). A minimum of 10,000 cells per sample were analyzed with Flow-Jo standard software (Version 10, TreeStar Inc., Ashland, OR, USA). Results are expressed as the mean apoptosis percentage of annexin-positive and propidium iodide-negative cells and double-positive cells.

### 4.10. Keratinosens^TM^ Assay

Keratinocyte activation by the Keap1/Nrf2-ARE (antioxidant response element) pathway was measured by the KeratinoSens^TM^ assay [48]. The assay uses an antioxidant response element (ARE)-coupled luciferase to sense Nrf2 activation. The KeratinoSens™ cell line was obtained from Givaudan (Vernier, Switzerland). The cells were cultured in DMEM supplemented with Glutamax^TM^, Fetal Calf Serum (FCS) 9.1%, and Geneticin^TM^ (500 μg/mL) at 37 °C in an atmosphere of 5% CO_2_ and 95% humidity. Cells were seeded on 96-well plates until reaching 80% confluency. Cells were then incubated with obacunone for 48 h in antibiotic-free DMEM. At the end of the incubation period, cells were washed and lysed for 20 min. Then, Promega firefly luciferase reagent was added, and the luminescence was immediately measured on a Lumistar plate reader (Lumistar Omega, BMG Labtech). An increase in luciferase activity in sample-treated cells was calculated in comparison to DMSO-treated cells (negative control) and expressed as fold luciferase activity induction (Imax). Following the manufacturer’s instructions, activation of the Nrf2 pathway was considered positive when the Imax was equal to or higher than 1.5 and statistically significant compared to the negative control.

### 4.11. Statistical Analyses

Results were expressed as mean ± standard error of the mean (SEM) of n experiments. Normal distribution for each data set was confirmed by the Kolmogorov–Smirnov test. Statistical analysis was carried out by multiple comparison analysis of variance (ANOVA) followed by the Bonferroni post hoc test. *p* < 0.05 was considered statistically significant. 

Correlations were expressed as Pearson’s correlation coefficients (rp). Correlation coefficients > 0.3 were considered to present positive correlations. Ranges between 0.5–0.7 and 0.7–0.9 were considered moderate and high correlation, respectively, while the range 0.9–1 was considered very high correlation [49]. Significance was inferred from a *p* value < 0.05.

## Figures and Tables

**Table 1:** Correlation between the solar-simulated radiation doses and the specific markers of photodamage. Correlation coefficients were calculated to assess the strength and direction of the linear relationships between pairs of variables. Correlations are expressed as Pearson’s correlation coefficient (r_p_). * *p* < 0.05.

	LDH	IL-1A	IL-6	IL-8	MMP1	MMP9
r_p_	0.99 *	0.91	0.88	0.93	0.89	−0.75
	ELN	DCN	COL1A1	COL7A1	P53	P21
r_p_	−0.69	0.61	−0.84	0.75	0.91	−0.97 *

## Data Availability

The datasets generated and/or analyzed during the current study are available from the corresponding author on reasonable request.

## References

[1] Roger M., Fullard N., Costello L., Bradbury S., Markiewicz E., O’Reilly S., Darling N., Ritchie P., Määttä A., Karakesisoglou I. (2019). Bioengineering the microanatomy of human skin. J. Anat..

[2] Sánchez-Marzo N., Pérez-Sánchez A., Ruiz-Torres V., Martínez-Tébar A., Castillo J., Herranz-López M., Barrajón-Catalán E. (2019). Antioxidant and Photoprotective Activity of Apigenin and its Potassium Salt Derivative in Human Keratinocytes and Absorption in Caco-2 Cell Monolayers. Int. J. Mol. Sci..

[3] Lawrence K., Al-Jamal M., Kohli I., Hamzavi I., Wang S.Q., Lim H.W. (2016). Clinical and Biological Relevance of Visible and Infrared Radiation. Principles and Practice of Photoprotection.

[4] Bernerd F., Vioux C., Asselineau D. (2000). Evaluation of the protective effect of sunscreens on in vitro reconstructed human skin exposed to UVB or UVA irradiation. Photochem. Photobiol..

[5] Marionnet C., Tricaud C., Bernerd F. (2014). Exposure to Non-Extreme Solar UV Daylight: Spectral Characterization, Effects on Skin and Photoprotection. Int. J. Mol. Sci..

[6] Schäfer M., Dütsch S., Auf dem Keller Keller U., Navid F., Schwarz A., Johnson D.A., Johnson J.A., Werner S. (2010). Nrf2 establishes a glutathione-mediated gradient of UVB cytoprotection in the epidermis. Genes Dev..

[7] Bernerd F., Marionnet C., Duval C. (2012). Solar ultraviolet radiation induces biological alterations in human skin in vitro: Relevance of a well-balanced UVA/UVB protection. Indian J. Dermatol. Venereol. Leprol..

[8] Meloni M., Farina A., de Servi B. (2010). Molecular modifications of dermal and epidermal biomarkers following UVA exposures on reconstructed full-thickness human skin. Photochem. Photobiol. Sci..

[9] Bernerd F., Asselineau D. (2008). An organotypic model of skin to study photodamage and photoprotection in vitro. J. Am. Acad. Dermatol..

[10] De la Vega M.R., Krajisnik A., Zhang D.D., Wondrak G.T. (2017). Targeting NRF2 for Improved Skin Barrier Function and Photoprotection: Focus on the Achiote-Derived Apocarotenoid Bixin. Nutrients.

[11] González S., Astner S., An W., Pathak M.A., Goukassian D. (2003). Dietary Lutein/Zeaxanthin Decreases Ultraviolet B-Induced Epidermal Hyperproliferation and Acute Inflammation in Hairless Mice. J. Investig. Dermatol..

[12] Nichols J.A., Katiyar S.K. (2009). Skin photoprotection by natural polyphenols: Anti-inflammatory, antioxidant and DNA repair mechanisms. Arch. Dermatol. Res..

[13] Wondrak G.T., Alberts D., Hess L.M. (2014). Sunscreen-Based Skin Protection Against Solar Insult: Molecular Mechanisms and Opportunities. Fundamentals of Cancer Prevention.

[14] Saw C.L., Huang M.-T., Liu Y., Khor T.O., Conney A.H., Kong A.-N. (2011). Impact of Nrf2 on UVB-induced skin inflammation/photoprotection and photoprotective effect of sulforaphane: Photoprotection of Nrf2 and sulforaphane. Mol. Carcinog..

[15] Xian D., Xiong X., Xu J., Xian L., Lei Q., Song J., Zhong J. (2019). Nrf2 Overexpression for the Protective Effect of Skin-Derived Precursors against UV-Induced Damage: Evidence from a Three-Dimensional Skin Model. Oxidative Med. Cell. Longev..

[16] Tao S., Justiniano R., Zhang D.D., Wondrak G.T. (2013). The Nrf2-inducers tanshinone I and dihydrotanshinone protect human skin cells and reconstructed human skin against solar simulated UV. Redox Biol..

[17] Rojo de la Vega M., Zhang D.D., Wondrak G.T. (2018). Topical Bixin Confers NRF2-Dependent Protection Against Photodamage and Hair Graying in Mouse Skin. Front. Pharmacol..

[18] Zhou J., Wang T., Wang H., Jiang Y., Peng S. (2019). Obacunone attenuates high glucose-induced oxidative damage in NRK-52E cells by inhibiting the activity of GSK-3β. Biochem. Biophys. Res. Commun..

[19] Bai Y., Wang W., Wang L., Ma L., Zhai D., Wang F., Shi R., Liu C., Xu Q., Chen G. (2021). Obacunone Attenuates Liver Fibrosis with Enhancing Anti-Oxidant Effects of GPx-4 and Inhibition of EMT. Molecules.

[20] Gao Y., Hou R., Liu F., Liu H., Fei Q., Han Y., Cai R., Peng C., Qi Y. (2018). Obacunone causes sustained expression of MKP-1 thus inactivating p38 MAPK to suppress pro-inflammatory mediators through intracellular MIF. J. Cell. Biochem..

[21] Huang D.-R., Dai C.-M., Li S.-Y., Li X.-F. (2021). Obacunone protects retinal pigment epithelium cells from ultra-violet radiation-induced oxidative injury. Aging.

[22] Liu Y., Wang R., He X., Dai H., Betts R.J., Marionnet C., Bernerd F., Planel E., Wang X., Nocairi H. (2019). Validation of a predictive method for sunscreen formula evaluation using gene expression analysis in a Chinese reconstructed full-thickness skin model. Int. J. Cosmet. Sci..

[23] Marionnet C., Bernerd F. (2016). Organotypic models for evaluating sunscreens. Princ. Pract. Photoprotection.

[24] Storey A., Rogers J.S., McArdle F., Jackson M.J., Rhodes L.E. (2007). Conjugated linoleic acids modulate UVR-induced IL-8 and PGE2 in human skin cells: Potential of CLA isomers in nutritional photoprotection. Carcinogenesis.

[25] Torricelli P., Fini M., Fanti P.A., Dika E., Milani M. (2017). Protective effects of Polypodium leucotomos extract against UVB-induced damage in a model of reconstructed human epidermis. Photodermatol. Photoimmunol. Photomed..

[26] Duval C., Schmidt R., Regnier M., Facy V., Asselineau D., Bernerd F. (2003). The use of reconstructed human skin to evaluate UV-induced modifications and sunscreen efficacy. Exp. Dermatol..

[27] Murphy M., Mabruk M.J.E.M.F., Lenane P., Liew A., McCann P., Buckley A., Flatharta C.O., Hevey D., Billet P., Robertson W. (2002). Comparison of the expression of p53, p21, Bax and the induction of apoptosis between patients with basal cell carcinoma and normal controls in response to ultraviolet irradiation. J. Clin. Pathol..

[28] Acute response of human skin to solar radiation: Regulation and function of the p53 protein—ScienceDirect. https://www.sciencedirect.com/science/article/pii/S1011134401002044?via%3Dihub.

[29] Hollmann G., Linden R., Giangrande A., Allodi S. (2016). Increased p53 and decreased p21 accompany apoptosis induced by ultraviolet radiation in the nervous system of a crustacean. Aquat. Toxicol..

[30] Chaturvedi V., Qin J.-Z., Stennett L., Choubey D., Nickoloff B. (2003). Resistance to UV-induced apoptosis in human keratinocytes during accelerated senescence is associated with functional inactivation of p53. J. Cell. Physiol..

[31] Lei X., Liu B., Han W., Ming M., He Y.-Y. (2010). UVB-Induced p21 degradation promotes apoptosis of human keratinocytes. Photochem. Photobiol. Sci..

[32] Gao Y., Chen A., Huang X., Xue Z., Cao D., Huang K., Chen J., Pan Y. (2015). The Role of p21 in Apoptosis, Proliferation, Cell Cycle Arrest, and Antioxidant Activity in UVB-Irradiated Human HaCaT Keratinocytes. Med. Sci. Monit. Basic Res..

[33] Marrot L., Planel E., Ginestet A.-C., Belaïdi J.-P., Jones C., Meunier J.-R. (2010). In vitro tools for photobiological testing: Molecular responses to simulated solar UV of keratinocytes growing as monolayers or as part of reconstructed skin. Photochem. Photobiol. Sci..

[34] Brennan M., Bhatti H., Nerusu K.C., Bhagavathula N., Kang S., Fisher G.J., Varani J., Voorhees J.J. (2003). Matrix metalloproteinase-1 is the major collagenolytic enzyme responsible for collagen damage in UV-irradiated human skin. Photochem. Photobiol..

[35] Keurentjes A.J., Jakasa I., van Dijk A., van Putten E., Brans R., John S.M., Rustemeyer T., van der Molen H.F., Kezic S. (2021). *Stratum corneum* biomarkers after *in vivo* repeated exposure to sub-erythemal dosages of ultraviolet radiation in unprotected and sunscreen (SPF 50+) protected skin. Photodermatol. Photoimmunol. Photomed..

[36] Dunaway S., Odin R., Zhou L., Ji L., Zhang Y., Kadekaro A.L. (2018). Natural Antioxidants: Multiple Mechanisms to Protect Skin From Solar Radiation. Front. Pharmacol..

[37] Pinto D., Trink A., Giuliani G., Rinaldi F. (2022). Protective Effects of Sunscreen (50+) and Octatrienoic Acid 0.1% in Actinic Keratosis and UV Damages. J. Investig. Med..

[38] Kahremany S., Hofmann L., Gruzman A., Dinkova-Kostova A.T., Cohen G. (2022). NRF2 in dermatological disorders: Pharmacological activation for protection against cutaneous photodamage and photodermatosis. Free. Radic. Biol. Med..

[39] Kawachi Y., Xu X., Taguchi S., Sakurai H., Nakamura Y., Ishii Y., Fujisawa Y., Furuta J., Takahashi T., Itoh K. (2008). Attenuation of UVB-Induced Sunburn Reaction and Oxidative DNA Damage with no Alterations in UVB-Induced Skin Carcinogenesis in Nrf2 Gene-Deficient Mice. J. Investig. Dermatol..

[40] Chaiprasongsuk A., Janjetovic Z., Kim T.-K., Jarrett S.G., D’Orazio J.A., Holick M.F., Tang E.K., Tuckey R., Panich U., Li W. (2019). Protective effects of novel derivatives of vitamin D3 and lumisterol against UVB-induced damage in human keratinocytes involve activation of Nrf2 and p53 defense mechanisms. Redox Biol..

[41] Ryšavá A., Vostálová J., Svobodová A.R. (2021). Effect of ultraviolet radiation on the Nrf2 signaling pathway in skin cells. Int. J. Radiat. Biol..

[42] Marionnet C., Pierrard C., Golebiewski C., Bernerd F. (2014). Diversity of Biological Effects Induced by Longwave UVA Rays (UVA1) in Reconstructed Skin. PLoS ONE.

[43] Marionnet C., Pierrard C., Lejeune F., Sok J., Thomas M., Bernerd F. (2010). Different Oxidative Stress Response in Keratinocytes and Fibroblasts of Reconstructed Skin Exposed to Non Extreme Daily-Ultraviolet Radiation. PLoS ONE.

[44] Afaq F., Mukhtar H. (2001). Effects of solar radiation on cutaneous detoxification pathways. J. Photochem. Photobiol. B Biol..

[45] Svobodová A., Vostálová J. (2010). Solar radiation induced skin damage: Review of protective and preventive options. Int. J. Radiat. Biol..

[46] Meewes C., Brenneisen P., Wenk J., Kuhr L., Ma W., Alikoski J., Poswig A., Krieg T., Scharffetter-Kochanek K. (2001). Adaptive antioxidant response protects dermal fibroblasts from UVA-induced phototoxicity. Free. Radic. Biol. Med..

[47] Qin D., Ren R., Jia C., Lu Y., Yang Q., Chen L., Wu X., Zhu J., Guo Y., Yang P. (2018). Rapamycin Protects Skin Fibroblasts from Ultraviolet B-Induced Photoaging by Suppressing the Production of Reactive Oxygen Species. Cell. Physiol. Biochem..

[48] OECD (2022). Test No. 442D: In Vitro Skin Sensitisation. https://www.oecd-ilibrary.org/content/publication/9789264229822-en.

[49] Mukaka M.M. (2012). Statistics corner: A guide to appropriate use of correlation coefficient in medical research. Malawi Med. J..

